# The Impact of Hope and Resilience on Multiple Factors in Neurosurgical Patients

**DOI:** 10.7759/cureus.849

**Published:** 2016-10-26

**Authors:** Devika Duggal, Amanda Sacks-Zimmerman, Taylor Liberta

**Affiliations:** 1 Neurological Surgery, Weill Cornell Medical College

**Keywords:** hope, resilience, mood and functioning, treatment outcomes, protective factors, stable psychological traits, neurosurgical outcomes, psychological distress, optimal functioning

## Abstract

The purpose of the present article is to outline and review the impact of stable psychological characteristics on the emotional and functional outcomes of neurosurgical patients. Neurosurgical patients face adversity as inherent to their diagnoses and, consequently, experience emotional distress. Despite commonalities in diagnoses, diverse outcomes are seen post-neurosurgery, which are influenced by psychological factors. Therefore, an understanding of neurosurgical patients’ behavior, thoughts, and feelings surrounding their diagnoses, informed by psychological concepts, is important for both neuropsychology and neurosurgery.

## Introduction and background

### Hope and resilience

Neurosurgery patients face multiple adversities within their illness, including both the physical impact of the disease (i.e., pain and discomfort) and treatment, as well as the psychological aspects of the knowledge of having a potentially life-threatening illness. Hope and resilience are both stable, psychological traits that can act as protective factors against adversity. Hope is an optimistic attitude of mind based on an expectation of positive outcomes. Hope is probably best conceptualized by Snyder [1] as “a positive motivational state that is based on an interactively derived sense of successful (a) agency (goal-directed energy) and (b) pathways (planning to meet goals)” [2]. While the agency component makes available the willpower and determination to achieve goals, the pathways component is responsible for creating alternative routes to pursue these goals. Hopeful individuals possess positive thinking that is reflective of a realistic sense of optimism [3] as well as the belief that they can produce routes to desired goals [4]. Such individuals perceive obstacles as challenges to overcome and are able to utilize their optimism to plan alternatives to achieve their end goal [5]. Studies have found that hope is positively correlated with life satisfaction and serves as a buffer against the impact of negative and stressful life events [6]. Thus, individuals high in hope tend to show better athletic, academic, occupational, and health outcomes [5]. Hope can exist even in the context of a life-threatening health condition and can perhaps lead to better outcomes, as will be the premise for the present article.

Resilience is an individual's ability to adaptively respond to hardship, stress, and adversity [7] and has been defined as the ability to “bounce back” from negative events without succumbing to despair [8]. Individuals who report high levels of resilience typically portray an optimistic outlook, positive emotionality, curiosity, and openness to new experiences [8]. These positive emotions, in turn, typically lead to constructive attitudes and behaviors [9]. High resilience helps individuals positively cope with uncertainty, conflict, and failure [9]. The ability to positively cope with adverse events allows resilient individuals to adapt to significant life changes and, consequently, function better as they are able to emerge from a challenge stronger, wiser, and more powerful. Researchers have found that resilience correlates strongly with health and longevity, success, interpersonal satisfaction, and happiness [10]. Hope and resilience are closely aligned constructs, as they both include a tendency towards maintaining an optimistic outlook in the face of adversity. 

## Review

### Impact of hope and resilience on mood and functioning

Possessing hope and resilience positively affect mood and functioning. Hope and resilience have been associated with better physical and mental health outcomes in undergraduates [11] as well as better health and well-being and lower psychological distress in adults [12-13]. In addition, Ryden, et al. found that resilience is a protective factor against depression and the impact of stress [14]. Additionally, both traits have been found to positively influence the quality of life as well as act as a buffer against the negative impact of stressors [15]. Studies have found that an inverse relationship exists between optimism and depressive symptoms [16] as well as between optimism and suicidal ideation [17]. These findings have been consistently observed across a number of studies. Specifically, Gooding, et al. [18] indicated that higher levels of hope countered the negative effects of depression on resilience related to emotional regulation. Additionally, patients who report higher levels of hope and resilience are less likely to show mood disturbances [19] and tend to possess higher self-esteem [20]. By contrast, low levels of hope have been associated with depression [21]. 

### Hope and resilience in the context of a medical illness

The optimistic attitude inherent in hopeful individuals plays a key role in successfully coping with a medical illness and its prognosis, as well as in improving health-related quality of life [22]. This is likely due to the strong relationship between hope, resilience, and mood. Many studies have shown that mood states have a direct impact on physical health. For instance, indices of positive mood are associated with biological health markers, such as immune system response, cortisol profiles, and cardiovascular function [23]. In contrast, negative emotional states tend to correlate with excessive somatic symptoms [24]. Giltay, et al. [25] noted that optimism predicted less probability of mortality in general and, in particular, of cardiovascular mortality in a population of older adults. A study conducted on an oncological population found that low levels of hope significantly predicted premature mortality in young patients with breast cancer [26]. Hopeful and optimistic patients with head and neck cancer showed greater survival rates a year after diagnosis when compared to pessimists [27]. Ironson, et al. [28] suggested that optimism, less avoidant coping strategies, and lower levels of depression positively influence the prognosis of patients diagnosed with AIDS. A review study found that resilience is associated with positive factors specifically linked to physical illness, such as self-care, adherence to treatment recommendations, illness perception, and physical exercise [29]. Resilience was also found to be associated with a higher quality of life and lower emotional distress as well as fatigue in cancer patients and survivors [30]. The aforementioned studies suggest that the relationship between hope, resilience, and mood may influence physical health by encouraging involvement in health-promoting behaviors [31]. 

### Psychological distress and treatment outcomes

Emotional distress is a commonly observed symptom in individuals who receive diagnoses that require neurosurgical intervention and is predictive of poor health-related behaviors, including smoking, alcohol use, fatigue, and sleep difficulties [32]. Individuals given a neurological diagnosis requiring surgical intervention are understandably more susceptible to psychological disturbances, such as anxiety and depression. The overwhelming level of stress accompanying neurological diagnoses contributes to individual feelings of helplessness and hopelessness, often leading to negative mood states. Diagnoses with a variable to negative prognoses are prevalent in the neurosurgical field, including epilepsy, cerebral aneurysms, tumors, moderate to severe brain injuries, and spinal cord degeneration. Additionally, neurosurgical intervention, despite being beneficial, can negatively impact mood and/or daily functioning. After the neurosurgical intervention, individuals often experience cognitive dysfunction and mood changes in varying degrees (including irritability, low mood, anxiety, diminished attention, and processing speed) [33]. The presence of these mood states and emotions, in turn, may lead to poor treatment outcomes, including a higher rate of mortality [34]. Psychological distress and depressive symptoms are observed in the face of diseases and are typically associated with poor self-care and management. For instance, a longitudinal study linked distress to poor medication adherence and self-management (through diet and exercise) in Type 2 diabetic patients [35]. Studies on brain tumor patients have shown that psychological distress contributes to the deterioration of the quality of life and is implicated in the morbidity and mortality of brain tumor patients [36-37]. While it remains unclear exactly how mood disturbances and poor outcomes are linked, it is possible that negative mood states negatively affect the motivation to engage in health-related behaviors to enhance recovery. 

### Hope and resilience as protective factors

Protective factors are circumstances or attributes that help individuals deal more effectively with stressful events. Hope and resilience can act as protective factors against the subjective experience of hardship, specifically of receiving a potentially adverse medical diagnosis. These traits may help individuals overcome and/or become proactive in the context of medical diseases by making individuals less susceptible to negative mood states, which negatively impact physical health. Several studies have examined the relationship between hope, resilience, and coping techniques in the context of medical illness.

Resilience recognizes the need to take both reactive and proactive measures when faced with hardship. Resilience ameliorates the destructive impact of negative or traumatic events and, through psychological elasticity, contributes to recuperation from these hardships. Resilience also proactively uses setbacks as opportunities for growth [38]. While hope promotes a generally positive outlook, resilience facilitates the thought processes that underlie flexibility, adaptation, and, at times, improvisation in situations that are typically marked by uncertainty. Both hope and resilience facilitate the search for meaning despite circumstances that do not lend themselves to rational and logical explanations [39].

Hopeful and resilient individuals are often characterized by positive attitudes and optimistic outlooks. Positivity and optimism, in turn, are associated with better mood states as well as decreased occurrences of stress-related illnesses and reduced use of medical services [40]. These adaptive traits help to replenish emotional resources, relieve potential suffering, and enhance positive coping methods [41]. Studies of individuals with cancer have suggested that resilience provides at least partial protection against emotional distress, leading to better emotional adjustment [42]. Other studies have indicated that aspects of hope and resilience, such as positive expectations and optimism, have predicted better health outcomes after heart transplantation and coronary bypass surgery [43-44]. Another study has shown that promoting positivity can help prevent relapse of depression during the residual phase, where the risk of relapse is particularly high [45]. 

### Promoting optimal functioning

Given the present literature, it is clear that there is a need to go beyond traditional efforts of resisting and/or recovering from a disease. A greater focus on psychological treatment, specifically the promotion and understanding of the conditions that promote hope and resilience, is critical. A review study found that the factors associated with and/or predictive of resilience are self-efficacy, self-esteem, internal locus of control, optimism, mastery, social support, hardiness, empowerment, acceptance, determinism, personal growth, social support, coping strategies, spirituality, cognitive appraisal, and a sense of coherence [32].

Rousseau [22] found that hope is augmented by strengthening interpersonal relationships, exploring one’s faith, and learning to control one’s symptoms. Another study found that individuals with high levels of hope reported significantly higher levels of personal adjustment and global life satisfaction [46]. Furthermore, these individuals reported significantly less emotional distress [46]. These findings suggest that efforts to increase individuals’ levels of hope and resilience may promote optimal functioning in multiple life domains and can be developed through psychological treatment. 

### Current investigation and future research directions

Neurosurgery patients face multiple adversities within their illness involving both the physical impact of the disease and treatment, as well as the psychological aspects of the knowledge of having a potentially life-threatening illness. Studies examining the impact of hope and resilience on mood and functional outcomes in neurosurgical populations are scarce. Bunevicius, et al. [47] suggest that psychological distress, which leaves one vulnerable to anxiety and depression, is prevalent in brain tumor patients. These factors contribute to deterioration in health-related quality of life and are associated with increased mortality of brain tumor patients [35, 48]. It is possible that characteristics of hope and resilience may help individuals cope with psychological distress effectively and, consequently, engage in more appropriate health-related behaviors.

To this end, the present authors, psychologists within the Weill Cornell Department of Neurosurgery, are investigating the impact of hope and resilience on emotional states, functionality, and disease prognosis in pre- and post-neurosurgical individuals. The authors are expecting to enroll approximately 70 participants who have recently received a neurosurgical diagnosis. These individuals will be given a battery of tests that measure their hope and resilience levels as well as their present emotional and functional states. The Adult Hope Scale (AHS) and Resilience Scale (RS) will be used to assess hope and resilience; the Beck Depression Inventory (BDI) and Beck Anxiety Inventory (BAI) will measure characteristic attitudes and symptoms of depression and anxiety; the Adaptive Behavior Assessment System, Second Edition (ABAS-II) will assess functionality in the domains of communication, community use, functional academics, home living, health and safety, leisure, self-care, self-direction, social, and work; finally, the Modified Rankin Scale (mRS) will measure the degree of dependency in the daily activities of individuals who have experienced a neurological disability. Approximately six months after participants receive neurosurgical treatment, they will be given the same battery, and changes in mood and functioning will be measured. The authors expect to find that individuals who possess characteristics of hope and resilience prior to neurosurgical intervention will be less vulnerable to negative mood states and more likely to engage in health-promoting behaviors that aid recovery.  

## Conclusions

There is a spectrum of human characteristics that affects the relationship between receiving a medical diagnosis and its impact on emotional state. More recently, there has been a growing interest in identifying protective factors that help such patients maintain emotional stability in the face of adverse events [49]. Therefore, measuring hope and resilience may help predict outcomes among people coping with significant medical conditions, which could have implications for providing extra support for those patients who are low in these characteristics. Increased knowledge and understanding of these innate characteristics and their relationship with emotional stability and coping can be integrated into supportive services or interventions (e.g., psychological counseling). From this, patients may experience increased quality of life.
